# Identification of Genes Discriminating Multiple Sclerosis Patients from Controls by Adapting a Pathway Analysis Method

**DOI:** 10.1371/journal.pone.0165543

**Published:** 2016-11-15

**Authors:** Lei Zhang, Linlin Wang, Pu Tian, Suyan Tian

**Affiliations:** 1 College of Life Science, Jilin University, 2699 Qianjin Street, Changchun, Jilin, China, 130012; 2 Department of Neurology, The Second Hospital of Jilin University, 218 Ziqiang Street, Changchun, Jilin, China, 130041; 3 Division of Clinical Research, The First Hospital of Jilin University, 71 Xinmin Street, Changchun, Jilin, China, 130021; Odense University Hospital, DENMARK

## Abstract

The focus of analyzing data from microarray experiments has shifted from the identification of associated individual genes to that of associated biological pathways or gene sets. In bioinformatics, a feature selection algorithm is usually used to cope with the high dimensionality of microarray data. In addition to those algorithms that use the biological information contained within a gene set as a priori to facilitate the process of feature selection, various gene set analysis methods can be applied directly or modified readily for the purpose of feature selection. Significance analysis of microarray to gene-set reduction analysis (SAM-GSR) algorithm, a novel direction of gene set analysis, is one of such methods. Here, we explore the feature selection property of SAM-GSR and provide a modification to better achieve the goal of feature selection. In a multiple sclerosis (MS) microarray data application, both SAM-GSR and our modification of SAM-GSR perform well. Our results show that SAM-GSR can carry out feature selection indeed, and modified SAM-GSR outperforms SAM-GSR. Given pathway information is far from completeness, a statistical method capable of constructing biologically meaningful gene networks is of interest. Consequently, both SAM-GSR algorithms will be continuously revaluated in our future work, and thus better characterized.

## Introduction

With the development of major pathway databases, e.g., the **Kyoto Encyclopedia of Gene and Genomes** (KEGG) [1] and **Gene Ontology** (GO) [2], the coordinated effect of all genes inside a pathway or gene set on a phenotype has been increasingly explored. These databases organize different types of biological pathway or gene set information and record co-expressed/co-regulated patterns. Consequently, many pathway or gene-set analysis methods have been proposed [3–11]. In this article, the phrases “gene set” and “pathway” are used interchangeably.

Feature selection is usually implemented to cope with the high dimensionality issue in bioinformatics [12]. It has been shown that when a feature selection method incorporates pathway knowledge, it has a better predictive power and more meaningful biological implication [8,13,14]. Supervised group LASSO method proposed Ma et al [15] is one of such methods. Briefly, this method consists of two steps. First, LASSO is used to identify relevant genes within each cluster/group. Then the method selects relevant clusters/groups using a group LASSO. In their work, the clusters are generated using a K-mean method, and thus are mutually exclusive. In reality, however, it is common to have a gene involving in many gene sets or pathways. An alternative way to account for pathway knowledge is suggested by [16]. In this algorithm, a pseudo-gene taking the average expression value of all genes inside a gene set is created to represent the whole gene set, and then the downstream analysis is conducted using those pseudo-genes. However, this method is incapable of selecting individual relevant genes.

A novel direction of gene set analysis was proposed by [17], which aims at further reduction of a significant gene set into a core subset. The reduction step to a smaller-sized core subset is essential towards understanding the underlying biological mechanisms. The proposed method by [17] was named as significance analysis of microarray-gene set reduction (SAM-GSR). The issue addressed by SAM-GSR is also of interest in a feature selection algorithm, which motivates us to carry out feature selection using the SAM-GSR algorithm.

Multiple sclerosis (MS) is the most prevalent demyelinating disease and the principal cause of neurological disability in young adults [18]. Currently, MS can only be confirmed using invasive and expensive tests such as magnetic resonance imaging (MRI). Therefore, researchers are searching for an easier and cheaper diagnosis of MS with the aids of other technologies such as microarray [19–21]. However, the number of microarray experiments on MS is limited and the sample sizes of those studies are predominately small [22]. Consequently, a feature selection algorithm that downsizes the number of genes under consideration to a manageable scale is highly desirable for the classification of MS samples.

As a part of the recently-launched Systems Biology Verification (sbv) Industrial Methodology for Process Verification in Research (IMPROVER) Challenge [23], MS sub-challenge targeted specifically on the utilization of gene expression data for the purpose of MS diagnosis. Among the challenge participants who ranked top in this sub-challenge, two used the methods accounting for pathway knowledge. First, Lauria [24] used Cytoscape [25] to construct two separate clusters/networks to discriminate MS samples from controls. Since the modeling parsimony is not a concern in this method, the resultant signature might be not applicable in the clinical setting. Second, Zhao et al [26] implemented the method by Chen et al. [16] and generated one pseudo-gene for each pathway by averaging expression values of all genes in that pathway. Then a logistic regression with elastic net regularization on those resulting pseudo features was fitted. This method was shown to be inferior to the regularized logistic regression model on individual genes.

In this paper, we apply SAM-GSR to MS microarray data to explore if SAM-GSR can be used for the purpose of feature selection. Also, we propose an extension to SAM-GSR that explicitly accomplishes feature selection.

## Materials and Methods

### Experimental data

We considered two microarray datasets in this study. The first one included chips from the experiment E-MTAB-69 stored in the ArrayExpress [27] repository (http://www.ebi.ac.uk/arrayexpress). All chips were hybridized on Affymetrix HGU133 Plus 2.0 chips. In this study, there were 26 patients with relapsing-remitting multiple sclerosis (RRMS) and 18 controls with neurological disorders of a non-inflammatory nature. The second dataset was provided by the IMPROVER MS sub-challenge, which is accessible on the project website (http://www.sbvimprover.com). It was hybridized on Affymetrix HGU133 Plus 2.0, and there were 28 patients with RRMS and 32 normal controls.

Gene sets were downloaded from the **Molecular Signatures Database** (MSigDB) [5]. We considered both c2 and c5 categories. The c2 category includes gene sets from curated pathways databases such as KEGG and those manually curated from the literature on gene expression. The current version (version 4.0) of MSigDB c2 category included 4,722 gene sets annotating on 11,844 unique genes. The c5 category includes 1,454 gene sets annotated by GO terms.

### Experimental data

Raw data of the first dataset (E-MTAB-69) were downloaded from the ArrayExpress repository, and expression values were obtained using the GCRMA algorithm [28] and normalization across samples was carried out using quantile normalization. The resulting expression values were on log_2_ scale. When there were multiple probe sets representing the same gene, the one with the largest fold change was chosen. Then the resulting expression values of 19,851 unique genes were fed into downstream analysis. Raw data of the second set were downloaded from the sbv challenge website, and were separately pre-processed in the same way.

### Statistical Methods

#### SAM-GSR

SAM-GSR is an extension of the SAM-GS algorithm [29], with an objective of identifying the core gene subset within each selected pathway. It consists of two steps: SAM-GS to select relevant pathways and the reduction step to obtain the core subset. In SAM-GS step, the following statistic, named as SAM-GS, is defined for gene set *j*,

$$SAMGS_{j}=\sum_{i=1}^{\left|j\right|}d_{i}^{2}\;,\;d_{i}=({\bar{x}}_{d}(i)-{\bar{x}}_{c}(i))/(s(i)+s_{0})$$

where d_i_ is the SAM statistic [30] of gene *i* and calculated for each gene for gene set *j*, ${\bar{x}}_{d}(i)$ and ${\bar{x}}_{c}(i)$ are the sample averages of gene *i* for the diseased and control group, respectively. Parameter s(i) is a pooled standard deviation and is estimated by pooling samples over two groups. s_0_ is a small positive constant used to offset the small variability in microarray expression measurements, and |j| represents the number of genes within gene set *j*. Basically, the SAM-GS statistic for a gene set is the L_2_ norm of SAM statistics over all genes within the gene set.

**Fig 1:**
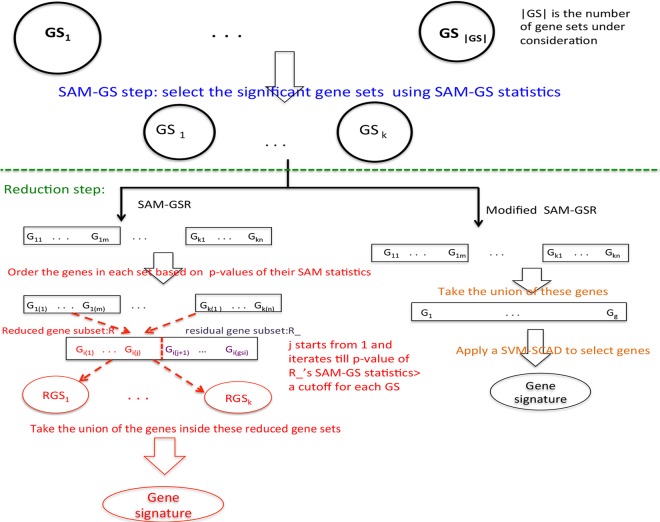
Graphical illustration of SAM-GSR and modified SAM-GSR algorithms. A. The SAM-GSR algorithm. B. The modified SAM-GSR algorithm.

Inside a significant gene set S, where its statistical significance is estimated using a permutation test by perturbing phenotype-labels for several hundred times, the reduction step gradually partitions the entire set S into two subsets: the reduced subset *R*_*k*_ and the residual one ${\bar{R}}_{k}$ for k = 1,…, |j|. After ordering genes in gene set *j* increasingly, based on the p-value of genes’ SAM statistics, the first k genes are enrolled into *R*_*k*_. Let c_k_ be the SAM-GS p-value of ${\bar{R}}_{k}$, the final size of *R*_*k*_ is set as the smallest k where c_k_ is larger than a pre-determined threshold for the first time. For more descriptions on the SAM-GSR algorithms, see the original work [17]. In addition, Fig 1A provides its graphical elucidation.

When using the SAM-GSR algorithm to execute feature selection, c_k_ can be regarded as a tuning parameter. Its optimal cutoff value is determined by conducting a sensitivity analysis in which a grid of values (i.e., 0.05 to 0.5 with an increment of 0.05) is considered. For each value, a support vector machine (SVM) [31] with the genes inside the resulting reduced subsets is fitted to calculate the misclassified error, i.e., the number of samples being falsely identified over the total sample size, on the training set. The optimal cutoff value of c_k_ is the one having the minimal misclassified error and the least number of selected genes. Lastly, we fit a SVM model upon the selected genes with c_k_ being set as the optimal cutoff, and evaluate the predictive performance of this final model using the test set.

#### Modified SAM-GSR

In SAM-GSR, whether a gene is selected into the core reduced subset *R*_*k*_ depends on the magnitude of its SAM statistic. It implies that if in a gene set |d_i_|> |d_k_| for genes *i* and *k*, gene *k* is possible to be involved in the reduce subset *R*_*k*_ only when gene *i* is in *R*_*k*_. When the goal is feature selection, however, the magnitude of individual SAM statistic might not matter so critically.

In this study, we propose to use a penalized machine learning method to perform feature selection and classify samples simultaneously. Because SVM is one of the widely used supervised learning methods, especially suitable for the two-class classification tasks of microarray data [32], we propose to use a SVM with a Smoothly Clipped Absolute Deviation (SCAD) [33,34] penalty to do feature selection. In a linear SVM model, the subjects from two distinct classes are separated by

$$f(x)=\sum_{i=1}^{G}w_{i}x_{i}+b$$

where x = (x_1_,..,x_G_) are the gene expression profiles, and x_i_ (*i* = 1,…G), a vector of length n, represents gene i’s expression profiles for n patients (n is sample size and G is the number of genes under consideration). And y (y = -1,1) is the class labels, w = (w_1_,…, w_G_) are the coefficients before gene expression values and represent the contribution of those genes to the hyperplane. A SVM model aims at finding the optimal hyperplane with maximal margin, which can be solved by,

$$\underset{b,w}{\min}{\sum \left(1-y_{i}f(x_{i})\right)}_{+}+pen_{\lambda }(w)$$

the above penalty term pen_λ_(w) is the sum of a SCAD penalty function over all coefficients, where the SCAD penalty function for coefficient *i* is defined by [34] as,

$$p_{\lambda }(w_{i})=\{\begin{array}{cc} \lambda |w_{i}| & if|w_{i}|\leq \lambda \\ -\frac{\left(|w_{i}{|}^{2}-2\alpha \lambda |w_{i}|+\lambda^{2}\right)}{2(\alpha -1)} & if\lambda <|w_{i}|\leq \alpha \lambda \\ \frac{(\alpha +1)\lambda^{2}}{2} & if|w_{i}|>\alpha \lambda \end{array}$$

where both α and λ are tuning parameters. For small coefficients, SCAD has the same behavior as L_1_/LASSO penalty [35], shrinking those coefficients to zeros. For large coefficients, however, its constant penalty produces smaller biases on the estimations. SVM-SCAD is implemented using R penalizedSVM package [36]. The default value for α is 3.7. Then for the grid of 2^−8^,2^−7^, 2^−6^, … and, 2^14^, λ is optimized via 5-fold cross validations (CV).

The procedure in which an SVM-SCAD model is implemented to select features, but restricting the genes under consideration to those inside the significant gene sets identified by SAM-GS, is referred to as modified SAM-GSR herein. Fig 1 elucidates graphically on both SAM-GSR and modified SAM-GSR algorithms.

### Statistical Metrics

Usually, using a single metric to evaluate an algorithm introduces biases. An algorithm may be erroneously claimed to be superior if a metric in favour of it is chosen or to be inferior if an unfavourable metric is used [23]. To avoid such biases, we used four metrics, namely, *Belief Confusion Metric* (BCM), *Area Under the Precision-Recall Curve* (AUPR), *Generalized Brier Score* (GBS), and error rate to evaluate the performance of a classifier.

Specifically, GBS is defined as using the equation by Yeung et al [37] and then dividing it by the sample size n,

$$GBS=\frac{1}{2n}{\sum_{i=1}^{n}\sum_{k=1}^{K}\left(Y_{ik}-p_{ik}\right)}^{2}$$

*where Y*_*ik*_ (1 if subject i belongs to class k, and 0 otherwise) are indicator functions for class k (*k* = 1,…,*K*), and *p*_*ik*_ denotes the predicted probability such that Y_ik_ = 1. GBS is in the internal of (0,1) while a value closer to zero indicates a better predictive. For more detailed description on GBS, see the work by [37,38].

BCM and AUPR are two metrics used by SBV challenge. As summarized by [39], BCM captures the average belief/confidence that a sample belongs to a class when indeed it belongs to this class. AUPR summarizes the ability of correctly ranking the samples known to be in a given class when sorted by the belief values decreasingly for that class. For these two metrics, the closer to 1 they are, the better a classifier is.

### Statistical language and packages

Statistical analysis was carried out in the R language version 3.1 (www.r-project.org), and R codes for SAM-GSR were downloaded from Dr. Yasui’s webpage (www.ualberta.ca/~yyasui/homepage.html).

## Results and Conclusions

**Fig 2:**
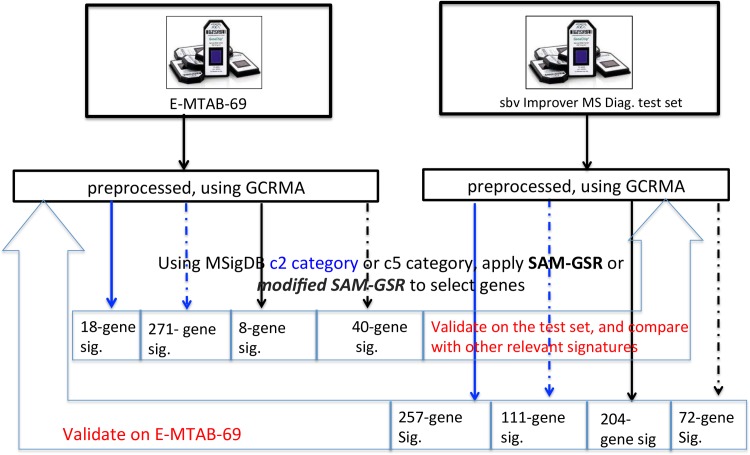
Study schema. Graphical illustration on how to analyze the multiple sclerosis (MS) microarray data.

**Fig 3:**
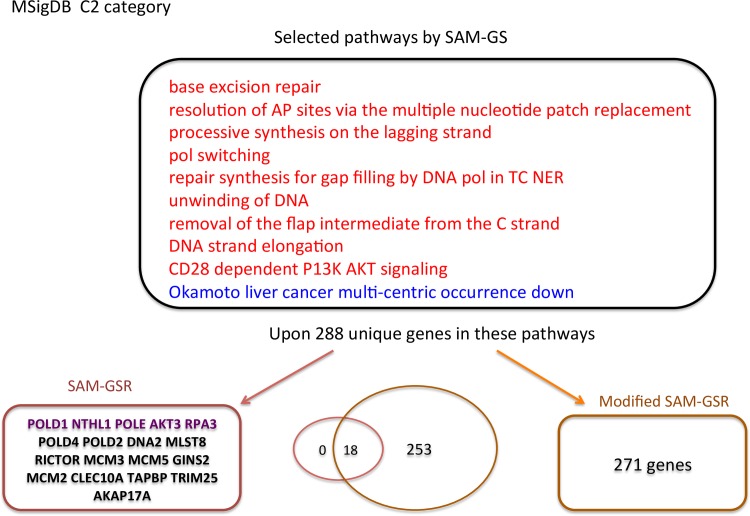
Selected pathways and genes by both SAM-GSR algorithms using pathways inside the MSigDB c2 category. Gene symbols in purple are the genes indicated as being directly related to MS by the GeneCards database. The overlapped gene symbols between the SAM-GSR and modified SAM-GSR algorithms are in bold.

**Fig 4:**
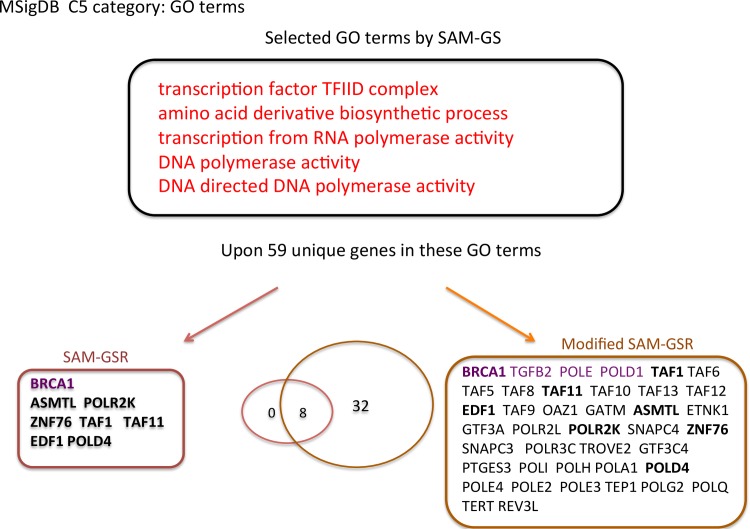
Selected pathways and genes by both SAM-GSR algorithms using pathways inside the MSigDB c5 category. Gene symbols in purple are the genes indicated as being directly related to MS by the GeneCards database. The overlapped gene symbols between the SAM-GSR and modified SAM-GSR algorithms are in bold.

The study schema is presented in Fig 2. First, we trained both SAM-GSR and modified SAM-GSR models on E-MTAB-69. The selected pathways and genes by both algorithms are provided in Figs 3 and 4.

To evaluate both algorithms, we computed their predictive statistics on the training (i.e., E-MTAB-69) and the test sets (i.e., the sbv test set). As shown in Table 1, the performance of modified SAM-GSR was superior to SAM-GSR on all performance statistics except for one AURP (0.612 versus 0.644, using the MSigDB c2 category). Then we reversed the order of these two datasets and reanalyzed them using the sbv MS test set as the training set. The performance statistics for the resulting signatures are given in Table 2. It is observed that the modified SAM-GSR algorithm outperforms the SAM-GSR algorithm with respect to both BCM and AUPR, e.g., the modified SAM-GSR achieves a BCM of 0.5 and an AUPR of 0.75 versus the SAM-GSR algorithm only has a BCM of 0.457 and an AUPR of 0.422, using the pathways in the MSigDB c5 category.

**Table 1:** Performance statistics of selected genes using E-MTAB-69 as the training set.

	E-MTAB-69				sbv Improver test set			
	Error (%)	GBS	BCM	AUPR	Error (%)	GBS	BCM	AUPR
C2: SAM-GSR (18)	20.45	0.121	0.701	0.896	46.67	0.464	0.500	0.644
C2: M-SAM-GSR (271)	0	0.066	0.747	0.992	46.67	0.291	0.520	0.612
C2: L1 as penalty (112)	0	0.083	0.719	0.992	33.33	0.207	0.564	0.776
C5: SAM-GSR (8)	13.64	0.134	0.673	0.904	46.67	0.464	0.500	0.579
C5: M-SAM-GSR (40)	0	0.046	0.800	0.992	43.33	0.365	0.577	0.703

Note: C2 represents the analyses using the pathways in MSigDB c2 category; C5 represents the analyses using the pathways in MSigDB c5 category. M-SAM-GSR abbreviates for modified SAM-GSR algorithm. GBS: Generalized Brier Score; BCM: Belief Confusion Metric; AUPR: Area Under the Precision-Recall Curve.

**Table 2:** Performance statistics of selected genes using the sbv Improver MS data as the training set.

	sbv Improver test set				E-MTAB-69			
	Error (%)	GBS	BCM	AUPR	Error (%)	GBS	BCM	AUPR
C2: SAM-GSR (257)	0	0.054	0.772	0.995	42.73	0.296	0.486	0.483
C2: M-SAM-GSR (111)	0	0.020	0.901	0.995	59.09	0.316	0.501	0.516
C5: SAMGSR (204)	0	0.046	0.793	0.995	54.55	0.337	0.457	0.422
C5: M-SAM-GSR (72)	0	<0.001	0.993	0.995	40.91	0.409	0.501	0.750

Note: C2 represents the analyses using the pathways in MSigDB c2 category; C5 represents the analyses using the pathways in MSigDB c5 category. M-SAM-GSR abbreviates for the modified SAM-GSR algorithm. GBS: Generalized Brier Score; BCM: Belief Confusion Metric; AUPR: Area Under the Precision-Recall Curve.

Interestingly, we observed that the model parsimony of the modified SAM-GSR algorithm suffers when trained on E-MTAB-69 while its parsimony is better than that of the SAM-GSR algorithm when trained on the sbv test set. We remark that when the SAM-GS statistic determines the significance level of a gene set, the decision of whether or not a gene is included in a reduced subset mainly depends on the magnitude of this gene’s SAM metric and the additive effect of genes in the reduced subset. Certainly, the number of gene sets in which a gene is involved also plays an important role. When a gene is involved in many gene sets, its likelihood of being selected increases several times compared to a barely isolated gene contained in only one or two gene sets. In contrast, such decision in the modified SAM-GSR algorithm hinges solely on genes’ contribution to the optimal hyperplane (i.e., weights) in the final SVM model.

Also in E-MTAB-69, the controls are those patients with neurological disorders of a non-inflammatory nature, such that the difference of expression values between MS and control in this data set is not as dramatic as the sbv test set in which the controls are normal individuals. After adjusting for the batch effect among different experiments using combat algorithm, the difference of expression profiles between a normal control and a control with non-inflammatory neurological disorders is distinct. This also explains why the predictive performance when trained on the sbv test set is not satisfying.

Therefore, we hypothesize that the modified SAM-GSR algorithm compromises on the model parsimony in order to obtain a good predictive performance when trained on E-MTAB-69. While the observation that the number of differentially expressed genes (DEGs) identified in the sbv test set is more than 10 times of that in E-MTAB-68 provides some support on this conjecture, further investigation is definitely needed.

### Comparison with other relevant signatures

We compared several MS diagnosis signatures in the literatures with the ones we obtained using both SAM-GSR algorithms. Here, we only compared the performance of different signatures on the sbv IMPROVER test set. The performance statistics of those signatures were tabulated in Table 3.

**Table 3:** Comparison with other relevant signatures on the sbv Improver set.

Study (size)	Training data used	Error (%)	GBS	BCM	AUPR
SAM-GSR (8)	E-MTAB-69	46.67	0.464	0.500	0.579
M-SAM-GSR (40)	E-MTAB-69	43.33	0.365	0.577	0.703
Lauria (n>100)	E-MTAB-69	—	—	0.884	0.874
Tarca (n = 2)	GSE21942 (on Human Gene 1.0 ST)	—	—	0.629	0.819
Zhao (n = 58)^a^	7 other data besides E-MTAB-69	30	—	0.576	0.820
Zhao (n = 84)^b^	7 other data besides E-MTAB-69	35	—	0.549	0.636
Tian (n = 28) ^1^	5 other data besides E-MTAB-69	68.33	0.546	0.345	0.362
Tian (n = 38) ^2^	E-MTAB-69	38.33	0.290	0.559	0.593
Guo (n = 8) *	E-MTAB-69	46.67	0.462	0.499	0.504

Note: M-SAM-GSR abbreviates for the modified SAM-GSR algorithm. GBS: Generalized Brier Score; BCM: Belief Confusion Metric; AUPR: Area Under the Precision-Recall Curve; —: not available.

* The predictive statistics on the test set for Guo’s study were calculated based on the 8-gene signature they provided in their article.

^1^The original submission by us to sbv IMPROVER using the TGDR algorithm, it was ranked around 30 among 54 participants.

^2^We trained TGDR on E-MTAB-69 to evaluate if different training sets result in difference performance of an algorithm.

^a^Zhao et al used elastic net to select individual genes, this submission ranked the third place in sbv MS subtask.

^b^Zhao et al used elastic net to select pseudo genes created by the averages of the genes inside pathways.

Most relevantly, Guo et al. [40] obtained an 8-gene signature using the same training set. This 8-gene signature ranked as the second worst, and only outperformed our original submission to sbv IMPROVER challenge. Compared with the top three teams in sbv MS diagnosis challenge, we remark that if we had submitted the results of modified SAM-GSR analysis to sbv IMPROVER challenge, we would have been ranked among top five.

In the worst performed signature, our original submission to the sbv challenge, the Threshold Gradient Descent Regularization (TGDR) [41] algorithm was utilized to conduct feature selection, and the training data sets included E-MTAB-69 in addition to five other microarray studies. Among these five microarray experiments, the chips from normal controls were included. Here, we reran TGDR analysis using E-MTAB-69 as the training set. The predictive performance improved dramatically, as indicated by the statistics in Table 3. There always exists data dependency for a feature selection algorithm [42]. Additionally, we think that the expression value profiles may be still be subject to batch effect even though we adjusted for it using combat algorithm [43]. Lastly, the distinct difference between normal controls and controls with other diseases might also play a role.

### Further verification using lung adenocarcinoma (AC) datasets

To further evaluate on both SAM-GSR algorithms, we applied these two algorithms to another set of real-world datasets. The objective is to discriminate histology stage I from stage II of lung adenocarcinoma patients. We trained both algorithms on a microarray dataset (GEO accession No: GSE 50081) and then evaluated the resulting signatures using 70 AC patients at early stages (i.e., stage I and II) in the RNA-seq data stored in The Cancer Genome Atlas (https://tcga-data.nci.nih.gov/tcga/). In this application, we only considered the pathways in the MSigDB c5 category.

For the RNA-seq data, Counts-per-million (CPM) values were calculated and log_2_ transformed by Voom function [44] in R limma package. For the microarray data, expression values were obtained using the fRMA algorithm [45], and then quantile normalization was carried out and those expression values were log_2_ transformed.

The results for both SAM-GSR algorithms in the AC application are given in Table 4. Moreover, we made a comparison of both SAM-GSR algorithms with three other feature selection algorithms, namely, SVM-SCAD, LASSO, and moderated t-test. These three algorithms are either well known in the field, e.g., LASSO or very relevant, e.g., SVM-SCAD. The performance statistics are presented in Table 4 as well. It is shown that modified SAM-GSR performs the best with respect to GBS and BCM, and SAM-GSR performs worse than SVM-SCAD in terms of predictive error, GBS, and BCM but ranks as the first in terms of AUPR. Overall, the modified SAM-GSR algorithm is the best among these five methods if all performance statistics are considered together.

**Table 4:** Performance statistics for the lung adenocarcinoma application.

Method	Size	TCGA RNA-Seq data			
		Error (%)	GBS	BCM	AUPR
SAM-GSR	111	35.7	0.357	0.5	0.692
M-SAM-GSR	89	44.3	0.312	0.552	0.666
SVM SCAD	117	32.9	0.329	0.54	0.645
Lasso	84	52.9	0.528	0.511	0.504
Moderated t-test	329	35.7	0.357	0.5	0.569

Note: M-SAM-GSR abbreviates for the modified SAM-GSR algorithm. GBS: Generalized Brier Score; BCM: Belief Confusion Metric; AUPR: Area Under the Precision-Recall Curve

## Discussion

The results of real-world applications show that the modified SAM-GSR algorithm has similar or better performance compared with the SAM-GSR algorithm and other novel feature selection algorithms. Moreover, the modified SAM-GSR algorithm has its distinguished merits. First, it requires less computational burden given it applies penalized SVM once instead of subsequently evaluating on SAM-GS statistics of the reduced subsets. Second, it automatically produces a final model that can be used to calculate a new sample’s posterior probability whereas SAM-GSR needs an extra application of SVM in order to obtain such probability.

To conclude, by incorporating the additional pathway knowledge contained in gene sets, both SAM-GSR algorithms have good performance, and they can be utilized for feature selection indeed. The modified SAM-GSR algorithm has advantages over the SAM-GSR algorithm. In the clinical setting, a feature selection algorithm that downsizes the number of genes to an understandable scale is imperative when using gene expression profiles for diagnostic purposes. Focusing on a smaller number of genes facilitates biological insight into disease processes and thus provides insight on the targeted therapies and intervention strategies. Furthermore, feature selection makes the replacement of a high-throughput microarray technology with some cheaper and quicker alternatives such as real-time PCR possible, thus increasing the applicability of the gene biomarkers in routine practice.

As indicated by the simulations in S1 File, both SAM-GSR algorithms have one drawback: when the true markers are only involved in few gene sets, both algorithms are highly unlikely to identify them. To alleviate or even eliminate this disadvantage, some specific modification on the SAM-GS step is needed. Moreover, the way of the SAM-GSR algorithms account for the pathway knowledge is obviously not seamless. Ignoring the pathway topology completely, the SAM-GSR algorithms heavily weigh on the number of gene sets inside which a gene is contained. Future study on these topics is warranted.

Given that pathway information is far from completeness, especially for an under-investigated disease such as MS, the de novo construction of biologically meaningful gene networks using a statistical method is recommended. The basic requirement for such a method is that it must take interactions and interplay among genes into account so that a gene is possible to appear in multiple gene sets. Then using the more appropriate and comprehensive pathway information, both SAM-GSR algorithms will be revaluated and better characterized.

## Supporting Information

S1 File Simulations to further evaluate on both SAM-GSR algorithms. (DOCX)
