# Supplementary material for: Identification of Genes Discriminating Multiple Sclerosis Patients from Controls by Adapting a Pathway Analysis Method
Source: PLoS One. 2016 Nov 15;11(11):e0165543. doi: 10.1371/journal.pone.0165543 (PMC5112852; doi:10.1371/journal.pone.0165543)
Supplement: S1 File — (DOCX) [file pone.0165543.s001.docx]

**File S1. Simulations**

Here, we conducted four simulations to evaluate both SAM-GSR algorithms on their feature selection capacity. Actual expression values of E-MTAB-69 data were used in simulations 1 and 2, and the sbv test dataset were used in the simulations 3 and 4, with an extra standardization to make the expression values of an individual gene have a mean of zero and a standard deviation of one.

In the first and third simulations, we chose the two genes selected by Tarca et al [1], i.e., F13A1 and GSTM1. These two genes are annotated within MSigDB c2 category, c5 category and hgu133plus2.db package. Then we randomly selected 998 genes, which served as noises, to make the total number of genes under consideration as 1000. The logit function for MS patients with controls as the baseline was given by,

$$f_{MSvsControl}=1.28X_{F13A1}-1.2X_{GSTM1}$$

the above coefficients of F13A1 and GSTM1 were simulated from an uniform distributed random variable in the range of -3 to 3. In this set of simulations, both genes are involved in multiple gene sets in both c2 (>40) and c5 (>20) categories.

In the second and fourth simulations, we chose two genes that are only involved in one or two gene sets in c2 and c5 categories as the relevant genes. The logit function for MS patients against controls was given as following,

$$f_{MSvsControl}=-(2.82X_{RP9}+2.41X_{COX4I2})$$

where the coefficients for RP9 and COX4I2 were simulated using an uniform distributed random variable in the range of -3 to 3, again.

The results for these four simulations are presented in Table S1. An important observation is that when the true marker appears in many gene sets, the SAM-GSR algorithm can identify it easily while the modified SAM-GSR is highly likely to miss it when trained on E-MTAB-69.

**Table S1. Performance of SAM-GSR and modified SAM-GSR on simulated data**

|  | SAM-GSR | | | Modified SAM-GSR | | |
| --- | --- | --- | --- | --- | --- | --- |
| A. Simulation 1, simulated using E-MTAB-69 | | | | | | |
|  | F13A1 | GSTM1 | Size | F13A1 | GSTM1 | Size |
| C2 | Yes | Yes | 13 | No | Yes | 35 |
| C5 | Yes | Yes | 14 | No | No | 42 |
| B. Simulation 2, simulated using E-MTAB-69 | | | | | | |
|  | RP9 | COX4I2 | Size | RP9 | COX4I2 | Size |
| C2 | No | Yes | 3 | No | Yes | 3 |
| C5 | No | Yes | 2 | No | Yes | 4 |
| C. Simulation 3, simulated using the SBV test set | | | | | | |
|  | F13A1 | GSTM1 | Size | F13A1 | GSTM1 | Size |
| C2 | Yes | Yes | 111 | Yes | Yes | 3 |
| C5 | Yes | Yes | 46 | Yes | Yes | 4 |
| D. Simulation 4, simulated using the SBV test set | | | | | | |
|  | RP9 | COX4I2 | Size | RP9 | COX4I2 | Size |
| C2 | No | No | 156 | No | No | 12 |
| C5 | No | Yes | 61 | No | No | 11 |

Note: Yes represents the gene is selected by algorithms; No means the gene is not selected; Size refers to the number of selected genes by SAM-GSR algorithms.

Another finding is the size of selected genes by the SAM-GSR algorithm is substantially smaller than that by the modified SAM-GSR algorithm when trained on E-MTAB-69 while the opposite is true when trained on the sbv test set. This is in consistent with the results of MS application. These two observations indicate the choice of a training set matters substantially in the process of machine learning. When trained on a dataset with more distinct expression values between two phenotypes and validated the constructed signature on a dataset with less distinct expression profiles, the performance of this signature is inferior to the signature constructed from the opposite cases.

Nevertheless, when the true markers are only involved in few gene sets, both SAM-GSR algorithms might miss them because their corresponding gene sets might not even pass the first step of SAM-GSR, namely, the selection on gene sets using SAM-GS. Another reason is that when a gene is involved in many gene sets, its probability of being selected increases. Obviously, the chance of this gene having a large enough SAM statistic in multiple gene sets is larger than that in a single gene set.

References:

1. Tarca AL, Than NG, Romero R: **Methodological approach from the Best Overall Team in the IMPROVER Diagnostic Signature Challenge**. *Syst Biomed* 2013, **1**:1–11.
